# *Thermoanaerosceptrum fracticalcis* gen. nov. sp. nov., a Novel Fumarate-Fermenting Microorganism From a Deep Fractured Carbonate Aquifer of the US Great Basin

**DOI:** 10.3389/fmicb.2019.02224

**Published:** 2019-09-27

**Authors:** Scott D. Hamilton-Brehm, Laura E. Stewart, Mavrik Zavarin, Matt Caldwell, Paul A. Lawson, Tullis C. Onstott, Joseph Grzymski, Iva Neveux, Barbara Sherwood Lollar, Charles E. Russell, Duane P. Moser

**Affiliations:** ^1^Division of Earth and Ecosystems Sciences, Desert Research Institute, Las Vegas, NV, United States; ^2^Department of Microbiology, Southern Illinois University Carbondale, Carbondale, IL, United States; ^3^Madison Area Technical College, Madison, WI, United States; ^4^Lawrence Livermore National Laboratory, Livermore, CA, United States; ^5^Department of Microbiology and Plant Biology, University of Oklahoma, Norman, OK, United States; ^6^Department of Geosciences, Princeton University, Princeton, NJ, United States; ^7^Department of Earth Sciences, University of Toronto, Toronto, ON, Canada; ^8^Division of Hydrologic Sciences, Desert Research Institute, Las Vegas, NV, United States

**Keywords:** 16S rRNA, subsurface, deep biosphere, *Firmicutes*, fractured carbonate, aquifer, borehole, Death Valley Regional Flow System

## Abstract

Deep fractured rock ecosystems across most of North America have not been studied extensively. However, the US Great Basin, in particular the Nevada National Security Site (NNSS, formerly the Nevada Test Site), has hosted a number of influential subsurface investigations over the years. This investigation focuses on resident microbiota recovered from a hydrogeologically confined aquifer in fractured Paleozoic carbonate rocks at 863 – 923 meters below land surface. Analysis of the microorganisms living in this oligotrophic environment provides a perspective into microbial metabolic strategies required to endure prolonged hydrogeological isolation deep underground. Here we present a microbiological and physicochemical characterization of a deep continental carbonate ecosystem and describe a bacterial genus isolated from the ecosystem. Strain DRI-13^T^ is a strictly anaerobic, moderately thermophilic, fumarate-respiring member of the phylum *Firmicutes*. This bacterium grows optimally at 55°C and pH 8.0, can tolerate a concentration of 100 mM NaCl, and appears to obligately metabolize fumarate to acetate and succinate. Culture-independent 16S rRNA gene sequencing indicates a global subsurface distribution, while the closest cultured relatives of DRI-13^T^ are *Pelotomaculum thermopropionicum* (90.0% similarity) and *Desulfotomaculum gibsoniae* (88.0% similarity). The predominant fatty acid profile is iso-C_15__:__0_, C_15__:__0_, C_16__:__0_ and C_14__:__0_. The percentage of the straight-chain fatty acid C_15__:__0_ is a defining characteristic not present in the other closely related species. The genome is estimated to be 3,649,665 bp, composed of 87.3% coding regions with an overall average of 45.1% G + C content. Strain DRI-13^T^ represents a novel genus of subsurface bacterium isolated from a previously uncharacterized rock-hosted geothermal habitat. The characterization of the bacterium combined with the sequenced genome provides insights into metabolism strategies of the deep subsurface biosphere. Based on our characterization analysis we propose the name *Thermoanaerosceptrum fracticalcis* (DRI-13^T^ = DSM 100382^T^ = ATCC TSD-12^T^).

## Introduction

Biomass in the continental crust may be greater than that on the surface of the Earth (Gold, 1992; Whitman et al., 1998; McMahon and Parnell, 2014; Magnabosco et al., 2018). The deep biosphere is an untapped reservoir of microbial biodiversity that may hold novel solutions to industrial, medical, and origin of life questions. While the concept of subsurface life was proposed in the 1920s (Bastin and Greer, 1930), research into deep terrestrial subsurface environments did not begin in earnest until the 1980s (Jorgensen, 2012). Exploration of subsurface microbial biogeochemical processes remain largely unmapped and uncharacterized, due partly to limited access to the terrestrial subsurface and the low biomass densities often found there. Primary sampling opportunities occur through caves, mines, springs, and boreholes. A high level of novel microbial diversity has been discovered in the subsurface through cultivation-independent sequencing of the 16S rRNA gene (Pedersen and Ekendahl, 1990; Kotelnikova and Pedersen, 1997; Takai et al., 2001; Chapelle et al., 2002; Moser et al., 2005; Lin et al., 2006; Osburn et al., 2014). However, only a modest fraction of known lineages are currently represented in culture collections (Daumas et al., 1988; Boone et al., 1995; Ravot et al., 1995; Nilsen et al., 1996; Puspita et al., 2012).

With some notable exceptions (Stevens and McKinley, 1995; Colwell et al., 1997; Fredrickson et al., 1997; Krumholz et al., 1997; Lehman et al., 2001; Osburn et al., 2014), the deep fractured rock ecosystems across most of North America have been relatively little studied for microbiology. However, the US Great Basin, in particular the Nevada National Security Site (NNSS, formerly the Nevada Test Site, Bowen et al., 2001), has hosted a number of influential subsurface investigations over the years (Amy et al., 1992; Haldeman and Amy, 1993; Haldeman et al., 1993; Kieft et al., 1997). Most of the NNSS lies entirely within a geologic extensional zone and is underlain by the “Death Valley Regional Flow System (DVRFS).” This expansive set of groundwater basins is dominated by fractured rock aquifers that comprises ∼100,000 km^2^ of mountain ranges (up to 3,600 m above mean sea level) and valleys which can reach below sea level (e.g., Death Valley, at −86 m, the lowest point in North America) (D’Agnese et al., 1997; Belcher et al., 2002; Ye et al., 2008; Bushmann et al., 2010). Within this system groundwater flows long distances (i.e., Interbasin Flow), from high-elevation recharge zones in central Nevada to large-discharge springs in and near Death Valley, CA, United States (Winograd and Thordarson, 1975; Winograd and Pearson, 1976; Belcher et al., 2009). Direct access to the DVRFS is achievable through an ongoing Department of Energy (DOE) sponsored environmental management activity tasked with tracking groundwater contamination associated with underground nuclear testing at the NNSS, the Underground Test Area (UGTA) sub-project. The hundreds of monitoring boreholes/wells established and maintained by this program represent a unique regional-scale observatory for deep life study.

This study focuses on resident microbiota in fluids from 863–923 meters below land surface (mbls), accessed via a monitoring borehole, U-3cn#5 (Garber and Johnson, 1967; Bangerter and Giblin, 1998). Intercepting a hydrogeologically confined aquifer within the fractured Paleozoic carbonate rocks of a prominent regional feature termed the “Lower Carbonate Aquifer” (LCA), this well provides a portal into a pristine deep ecosystem that would otherwise be very difficult to access. Analysis of the microorganisms living in this restricted oligotrophic environment provides a perspective into microbial metabolic strategies required to endure prolonged hydrogeological isolation deep underground. The isolation of this unit is demonstrated by the very low radiocarbon content (4.65 pmc ^14^C) and absence of contamination related to past nuclear testing in the overlying units. Here we present a microbiological and physicochemical characterization of one window into the NNSS portion of the DVRFS and describe the isolation of a representative bacterium from this ecosystem, DRI-13^T^, a novel, moderately thermophilic, strict fumarate-respiring organism belonging to the phylum *Firmicute*s from the deep terrestrial biosphere, here designated *Thermoanaerosceptrum fracticalcis*.

## Materials and Methods

### Field Site and Sample Collection

**FIGURE 1:**
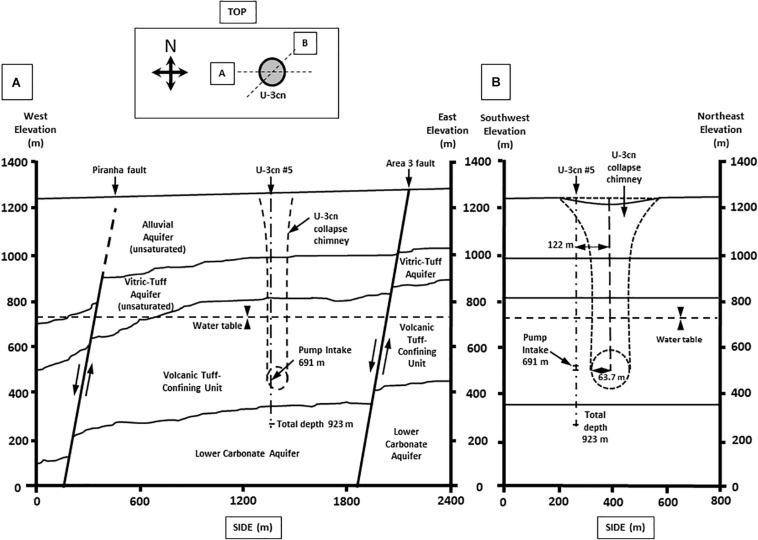
Geological cross section of well U-3cn #5. Cross section from **(A)** West to East and **(B)** Southwest to Northeast. Cross sections show borehole U-3cn#5 pump position, Total depth (TD) at 923 m, and relative location below the water table. (Image modified from McCall, RL, Raytheon Services Nevada, 1994, DOE/NV/-500-UC-700) [DOE cleared].

Well U-3cn#5 (Garber and Johnson, 1967; Bangerter and Giblin, 1998) is located in central Yucca Flat, Nevada, United States on the NNSS (latitude 37.06, longitude −116.02, surface elevation 1223 m). Yucca Flat is an arid, intermontane valley partially filled with tuffs and alluvium (260 – 290 m thickness), underlain by partially welded and zeolitized Tertiary-age ash-flow tuffs and bedded tuffs (∼536 m thickness). A highly fractured Paleozoic carbonate layer (colluvium transitioning to dolomite/dolomitic quartzite) of about 41 m thickness completes the sequence and is the source of groundwater for this study. The borehole was drilled in 1965 to a total depth of 923.5 mbls, 120 m southwest of surface ground zero and outside the collapse chimney of the Bilby underground nuclear test conducted in 1963 (249 kt, 63.7 m cavity radius) (Zavarin, 2014) (Figures 1A,B). The hole intersects the local groundwater table at 493.9 mbls and carbonate rocks at 863.2 mbls. Borehole gamma logs conducted within U-3cn#5 indicated the presence of elevated radionuclides within the unsaturated zone tertiary volcanic rock. These radionuclides were thought to have been emplaced by prompt injection during the underground test. Radionuclides have not been detected in the water-saturated fractured carbonates intersected by U-3cn#5, indicating no impact by the nearby underground nuclear test (Thompson, 1999). This volcanic confining unit overlying the carbonate aquifer serves to hydrogeologically isolate a nearly anoxic, slightly geothermal (∼45^o^C) portion of the LCA. The well is continuously cased through the vadose zone and confining unit (stainless steel below the water table) and was recompleted in 1996/1997, when the permanent 50-horsepower tandem pump used for this study was installed at 691.3 mbls. During pumping, water is obtained from an isolated open hole segment (863.2–923.5 mbls) within the LCA below the casing terminus; most likely derived from distinct fractured zone in core logs at 863.5, 864.4, and 866.2 mbls or a highly fractured zone from 868.7 to 876.3 mbls (Bangerter and Giblin, 1998).

Prior to sampling, U-3cn#5 was pumped and sampled on a semi-regular basis since 1967 (Total of 3 × 10^6^ m^3^ of water), including March 29, 2011, the subject of this report. Water samples were obtained on the surface from an in-line sampling port affiliated with a hydrologic pump test (104.9 gpm) after removal of 440 m^3^ of water over a period of days via an autoclaved gas-tight manifold. Water samples for microbial cultivation and dissolved gas sampling were transferred from flowing lines (LS-24 Nuprene Tubing, Masterflex) fitted with 24 Ga needles into sterile crimp-sealed, pre-evacuated, N_2_ flushed, 120 mL serum bottles (gas samples fixed with 5 μL of saturated HgCl_2_). Samples for aqueous chemistry were collected from the flowing sample lines and analyzed offsite. Samples for microbial community analysis were collected in two sterile 8 L polypropylene carboys and cells collected offsite on 0.2 μm filters (Sterivex, EMD Millipore, Bedford MA, 2 L per filter).

### Physical and Geochemical Analysis

Field measurements of temperature, dissolved O_2_ (dO_2_), conductivity, and pH were made onsite with an MP20 Sonde fitted with a flow cell (Yellow Springs Instruments, Yellow Springs, OH). Most chemical analytes were obtained during routine analysis by the DOE Underground Test Area (UGTA) Program. Additional analyses were performed at the Desert Research Institute (DRI) Water Analysis Laboratory (Reno, NV, United States) or at Princeton University (Princeton, NJ, United States). Nitrite, nitrate, and ammonia were analyzed at DRI using Alpkem RFA 300 and Technicon Automated Colorimetric Analyzers using EPA method SM 4500-NO_3_ F and SM 4500-NH_4_ F. Dionex Model ICS 2000 Ion Chromatograph was used for the measurement of Cl^–^, Br^–^, and SO_4_^2–^. A Brinkmann Metrohm Titrando automated titrator, capable of potentiometric titrations to fix inflection end points, was used in the determination of CO_3_^2–^, HCO_3_^–^, pH, EC, and F^–^. An OI Analytical 1030W Carbon Analyzer was used for determination of dissolved organic carbon (DOC) and total organic carbon (TOC) in water samples. A Thermo Elemental SOLAAR M5 Atomic Absorption Spectrometer with air-acetylene flame and vapor generation capabilities was used for major cation and many metal analyses.

A subset of samples were analyzed in duplicate for anions (F^–^, Cl^–^, NO^2–^, SO_4_^2–^, Br^–^, NO_3_^–^, PO_4_^3–^) and for short chain fatty acids (acetate, lactate, formate, and propionate) at Princeton University using a Dionex IC25 ion chromatograph (Thermo Scientific, Waltham, MA, United States) coupled to an MSQ Plus^TM^ ESI-quadrupole mass spectrometer (Thermo Scientific, Waltham, MA, United States) (Lau et al., 2014).

Total and Live/Dead cell counts were determined for groundwater samples using mfgr-provided reagents and protocols with a MicroPro Flow Cytometer (Benton Dickenson).

### Environmental DNA and Clone Libraries

Planktonic microorganisms, concentrated by filtration, were extracted using the UltraClean^TM^ Soil DNA Isolation kit (Mo Bio Laboratories Inc., Carlsbad, CA, United States), according to manufacturer’s protocol amended with three freeze/thaw cycles (−80°C/65°C; 20 min each) at the beginning of DNA extraction procedure. Given the proximity of the site to prior nuclear testing, filters were screened for radioactivity by scintillation at the Harry Reid Center for Environmental Studies at the University of Nevada, Las Vegas as a precaution (all were below detection limits). The extracted DNA was used for PCR-amplified clone libraries, universal bacterial primers 27F-YM/149 2R (AGAGTTTGATYMTGGCTCAG/TACCTTGTTACGACTT) and universal archaeal primers were used 21F/149 2R (TTCCGGTTGATCCYGCCGGA/TACCTTGTTACGACTT) to generate near-full-length 16S rRNA gene amplicons (Lane, 1991). PCR was performed using LATaq (Clontech, Mountain View, CA, United States) and thermocycler settings of 95°C, 30 s; 53°C, 30 s; 72°C, 60 s; for 30 cycles. PCR amplicons were purified with an UltraClean GelSpin DNA Purification Kit (Mo Bio Laboratories Inc., Carlsbad, CA, United States), cloned (TOPO-TA, Invitrogen, Carlsbad, CA, United States) and contigs were completely sequenced for bacteria and partially sequenced for archaea by Functional BioSciences (Madison, WI, United States) using vector primers. Contigs were generated using Sequencher^TM^ 4.9 (Gene Codes, Ann Arbor, MI, United States), aligned, matched with nearest neighbors and checked for chimeras using Silva (v1.2.11) (Glöckner et al., 2017) and MEGA 5.2 (Tamura et al., 2011).

### Enrichment Cultivation and Isolation

Microbial cultivation enrichments were performed in 160 mL serum bottles with 25 mL of a custom artificial groundwater medium (AGM), composed of per liter 3.6 g 2-[4-(2-hydroxyethyl)piperazin-1-yl]ethanesulfonic acid (HEPES), 1.5 g Na_2_SO_4_, 0.174 g K_2_PO_4_, 0.138 g Resazurin, 0.4 g MgCl_2_6 H_2_O, 0.5 g KCl, 0.268 g NH_4_Cl, 0.25 g NaHCO_3_, 1 mL ATCC Minimal Vitamins (ATCC, Manassas, VA, United States), and 1 mL ATCC Minimal Minerals. AGM was prepared anaerobically using a modified Hungate technique with 600 mg/L Na_2_S⋅9H_2_0 as a reducing agent (Miller and Wolin, 1974). The water sample was maintained at 4°C for 2 years prior to attempting enrichments. The primary enrichment consisted of 1 mL inoculum from the environmental sample, incubated in AGM amended with 0.1% (w/v) peptone (BD Bacto, Franklin Lakes, NJ, United States) at 45°C with a headspace of 100% N_2_. Cell numbers were monitored using a Petroff-Hausser counter (Hausser Scientific Co., Horsham, PA, United States) and an Axioskop2 Plus microscope under phase contrast. Cells reached a density of 10^6^ cells/mL after 1 month and were subsequently transferred three times prior to final isolation by low-intensity heat shock, serial dilution, and streak plating. Heat shocks were performed first on the environmental enrichment by rapidly raising the incubation temperature during logarithmic growth from 45°C to 60°C for 1 h and then rapidly cooling to 45°C. This was repeated for three transfers, the resulting cultures were then serial diluted to sub single cell concentrations. Finally, the putative isolate was streaked on anaerobic 1% w/v agar plates (AGM with agar added) amended with 10 mM fumarate/0.05% (w/v) yeast extract and one of the apparently identical colonies was selected as the type strain.

### Cell Growth Assays

Growth experiments were conducted in 160-mL serum bottles, containing 25 mL volume of AGM and 10 mM fumarate with a headspace of 100% N_2_. Cultivation experiments to define temperature optima were incubated at 37, 45, 50, 55, 60, and 65°C in the dark without shaking. Cultivation experiments to determine optimal pH utilized alternative buffers, replacing the HEPES buffer when not appropriate. A final concentration of 10 mM for each buffer was used to achieve the desired pH value. The buffers used were: 1,4-Piperazinediethanesulfonic acid (PIPES) for pH 6.5-7.5, HEPES for pH 8-8.5, and 3-([1,1-Dimethyl-2-hydroxyethyl]amino)-2-hydroxypropanesulfonic acid (AMPSO) for pH 9. All culturing optimization tests were completed in quadruplicate.

Substrate utilization was determined by transferring strain DRI-13^T^ three times at 55°C into AGM media containing 10 mM of the following defined substrates: glucose, formate, fumarate, lactate, acteate, pyruvate, methanol, propionate, ribose, xylose, oxaloacetate, succinate, tartarate, butyrate, malate, citrate, aspartate, methionine, valine, alanine/glycine, leucine/glycine, and glutamate/glycine. Undefined substrates were added to AGM media in concentrations of 0.1% wt/vol for casamino acids, peptone, and yeast extract. Gas phase substrates were added to the serum bottle headspace to a pressure of 30 psi for H_2_/CO_2_ (80:20) and CO (100). Electron acceptors 5 mM nitrite, 5 mM nitrate, 10 mM sulfate, 10 mM sulfite, 10 mM thiosulfate, and 0.01% wt/vol elemental sulfur were individually added to AGM medium with each defined and undefined substrate. Success was determined when cultures were successfully transferred three times and reached cell densities of 10^8^ cells/mL by microscopy observations using a Petroff-Hauser cell counting chamber.

Co-culture experiments of strain DRI-13^T^ with wild type *Methanothermobacter thermoautotrophicum* were conducted in AGM medium containing 10 mM fumarate or 30 psi H_2_/CO_2_ (80/20 headspace) at 55°C. Enumeration of both microorganism’s growth was monitored by microscopy based on their distinct morphologies.

Fumarate depletion was monitored by high-performance liquid chromatography (HPLC). Supernatants were filtered and acidified with 200 mM sulfuric acid to a final concentration of 5 mM. A sample of 5 μL was injected into AcclaimTM OA 5 μm column (4 × 250 mm), (Thermo Scientific), housed in Agilent 1100 series HPLC system for each time point. Separation was achieved under an isocratic gradient at a temperature of 38°C and flow of 0.5 mL/min. The DAD detector was adjusted to a wavelength of 210 nm and full scale sensitivity. Retention times of obtained peaks were compared with known standards (Organic Acids Kit, Supelco Analytical) and the quantity calculated using linear standard curves. The results were assessed with Chromeleon 7 software.

### 16S rRNA Gene Sequence Analysis and Genomic DNA Extraction

Strain DRI-13^T^ isolate was identified by centrifuging 5 mL of planktonic cells during logarithmic growth at 10,000 × *g* for 15 min at 4°C. DNA was extracted from the resulting cell pellet using an UltraClean Microbial DNA Isolation Kit (Mo Bio, Carlsbad, CA, United States). Universal bacterial primers 27F/1492R (5′-AGAGTTTGATCMTGGCTCAG-3′/5′-ACCTTGTTACGACTT-3′) were used to PCR amplify the small subunit ribosomal RNA (SSU rRNA or 16S rRNA gene) (Weisburg et al., 1991). The PCR product was then sequenced by Functional Biosciences (Madison, WI, United States) to determine identification of the isolate. With other 16S rRNA gene sequences recovered from the National Center for Biotechnology Information (NCBI) website, the sequences were aligned and phylogenetic relationships determined by Silva (v1.2.11) (Glöckner et al., 2017) and MEGA 5.2 (Tamura et al., 2011). Taxonomic classification sequences were obtained from the “List of Prokaryotic names with Standing in nomenclature” (LPSN,^1^).

High-molecular weight DNA was extracted from DRI-13^T^ by cultivating the organism in five, 1-L batch cultures grown on 10 mM fumarate. Cells were harvested by centrifugation at 15,000 × *g* for 30 min, and DNA was precipitated and extracted by cetyltrimethyl ammonium bromide (CTAB) buffer/phenol/chloroform (Sambrook and Russell, 2001). DNA was RNase treated, quality-checked on 1% agarose gel and shipped to the DOE Joint Genome Institute (JGI) for draft sequencing using Illumina and PacBio sequencing technology (PacBio RS; Illumina HiSeq 2500). The sequences were assembled according to JGI protocols using Velvet, annotated using a suite of gene characterization tools, and made available as a part of the JGI-IMG data warehouse (Markowitz et al., 2014). The genome of DRI-13^T^ was released to the public as per JGI protocol and is available both through the DOE IMG platform and via NCBI (Accession# PRJNA234897). The full length 16S rRNA gene sequence was submitted to NCBI and assigned accession number KR014122.

### Electron Microscopy and Sample Preparation

For SEM, the samples were moved to a 13 mm Swinney micofilter holder with 0.2 μm Millipore filters and dehydrated. Dehydration of the cells was done with 25% increases of ethanol for 15 min each step from 25 to 95%, then three washes of 100%. Once dehydrated, the filters were critical point dried with a Tousimis Samdri CPD (Tousimis, Rockville MD, United States), mounted on aluminum stubs and coated with approximately 25 nm layer of gold with a SPI sputter coater (SPI supplies, West Chester, PA, United States). Images were taken on a Zeiss 1450EP SEM at 10 kV.

Cells for transmission electron microscopy (TEM) and scanning electron microscopy (SEM) were aseptically removed from anaerobic serum bottles and immediately fixed with 2% v/v glutaraldehyde in 0.1 M sodium cacodylate buffer for 1 h at 4°C. Cells were pelleted by centrifugation (10,000 × *g* for 10 min) between all steps and then re-suspended each time. The sample was then rinsed with 0.1 M sodium cacodylate buffer for 10 min each for three times and re-suspended in 1% OsO_4_ in 0.1 M sodium cacodylate buffer for an hour at 4°C, then rinsed in distilled water for 10 min twice. TEM dehydration of the cells was performed with 25% increases of ethanol for 15 min each step from 25 to 95%, then three washes of 100%. Samples were then infiltrated with Spurr’s low viscosity resin (EMS, Hatfield, PA, United States), with increasing concentrations of 100% ethanol and resin. After three changes of 100% resin and 1 h between each change, the samples were then placed in a 70°C oven for polymerization for 12 h. The resin was cut into 50–70 nm thick sections on a MT-X ultramicrotome (RMC, Tuscon, AZ, United States) and images were obtained on a JEOL 1011 TEM (JEOL, United States, Peabody, MA, United States) operating at 80 kV and equipped with an AMT camera system.

### Lipid Analysis

For analysis of cellular fatty acids, biomass was harvested from cells grown in fumarate-amended (10 mM) mineral media for 72 h at 55°C. Analysis was performed at the Center for Microbial Identification and Taxonomy (University of Oklahoma). Fatty acid methyl esters were extracted, separated, and analyzed using the Sherlock Microbial Identification System (MIDI) version 6.1 as described previously (Kampfer and Kroppenstedt, 1996; Sasser, 2001). Analysis was performed with an Agilent Technologies 6890N gas chromatograph equipped with a phenyl methyl silicone fused silica capillary column (HP-Ultra 2, 25 m × 0.2 mm × 0.33 μm film thickness) coupled with a flame ionization detector. Hydrogen was used as the carrier gas. The temperature program was preset at 170°C and increased at 5°C min^–1^ to a final temperature of 270°C. The identification and relative abundance of each fatty acid was expressed in terms of the percentage of total fatty acids using the QTSA1 database.

## Results

### Hydrogeological Setting

The physiological and geochemical characteristics of the water samples from the pump test of U-3cn#5 on March 29, 2011 are presented in Table 1. Borehole water was fresh (845 μS/cm or 540 ppm TDS) (Kang and Jackson, 2016), slightly alkaline (pH 7.7), and was 44.7°C, as recorded at the surface. The dissolved oxygen (DO) was measured at 0.19 mg/L (3.2% of saturation), with no detectable sulfide. Reduction/oxidation potential was not measured. Total inorganic carbon (TIC) was 50.3 mg C/L, which at this pH would have been mostly bicarbonate. Major ions were defined by sodium > calcium > sulfate > silica (53.5, 37.2, 36, and 35 mg/L, respectively), followed by lesser amounts of chloride, magnesium, and potassium. Total metals were dominated by iron (1.57 mg/L), followed by lesser amounts of strontium (248 μg/L), manganese (101 μg/L), zinc (10.9 μg/L), and molybdenum (4.8 μg/L). Inorganic nitrogen was dominated by ammonium and nitrate, which were detected at 0.05 mg/L and 0.26 mg/L; while nitrite was below the 0.01 mg/L detection limit. Phosphate was detectable at 0.033 mg/L. Total organic carbon (TOC) was present at 1.5 mg/L, a substantial proportion of which is represented by the short chain fatty acids formate, acetate, and lactate, measured at 0.65, 0.4, and 0.4 mg/L, respectively. Propionate and fumarate were below detection limits. Tritium, a marker for the impact of nuclear device testing, was <7 pCi/L. Stable isotope signatures of d^13^C for dissolved inorganic carbon (DIC) of −12.8‰, and d ^2^H of -108‰ were detected as well.

**TABLE 1:** Physical and chemical characteristics of source water from borehole U-3cn#5.

Latitude	37.06	Mn (μg/L)	101
Longitude	−116.02	Mo (μg/L)	4.8
Depth of sample (m)	863–923	Zn (μg/L)	10.9
Total Depth of well (m)	924	Cu (μg/L)	<0.09
Sampling method	Pump	Cr (μg/L)	<0.18
Temperature (°C)	44.7	Ni (μg/L)	2.6
pH	7.69	U (μg/L)	0.83
Rock type	carbonate	Pb (μg/L)	0.072
DO (mg/L)	0.193	Sr (μg/L)	248
DO (%Sat.)	3.2	Cs (μg/L)	2.11
^3^H (pCi/L)	<6.5	W (μg/L)	0.49
TOC (mg C/L)	1.5	Acetate (mg/L)	0.4
TIC (mg C/L)	50.3	Lactate (mg/L)	0.4
Live/Dead Cell counts	4245	Formate (mg/L)	0.65
Total cell counts	10887	Propionate (mg/L)	<0.01
Conductivity (μS/cm)	845	Fumarate (mg/L)	<0.01
Turbidity (NTU)	3.675	He (% vol/vol)	<0.01
SO_4_^2–^ (mg/L)	36	H_2_ (% vol/vol)	<0.01
NO_3_^–^ (mg/L)	0.26	O_2_ (% vol/vol)	17.1
NO_2_^–^ (mg/L)	<0.14	N_2_ (% vol/vol)	65.0
NH_4_^+^ (mg/L)	0.05	CO_2_ (% vol/vol)	16.9
PO_4_^3–^ (mg/L)	0.033	CH_4_ (% vol/vol)	0.17
SiO_2_ (mg/L)	35	C_2_H_6_ (% vol/vol)	<0.01
Cl (mg/L)	27.4	C_3_H_8_ (% vol/vol)	<0.01
Na (mg/L)	53.5	iso-C_4_H_10_ (% vol/vol)	<0.002
K (mg/L)	8.5	n-C_4_H_10_ (% vol/vol)	0.002
Ca (mg/L)	37.2	d^13^C CO_2_ (in ‰ V-PDB)	−12.8
Mg (mg/L)	16.5	d^13^C Methane (in ‰ V-PDB)	b.d.
Br (mg/L)	0.09	d ^2^H (‰)	−108
Fe (mg/L)	1.57	^14^C (pmc)	4.65

*b.d., below detection limit of instrument.*

### Indigenous Microbial Communities

Cell enumeration indicated a total planktonic cell density in borehole water of 1.1 × 10^4^ cells/mL. Of these, a substantial proportion (∼half, 4.2 × 10^3^ cells/mL) were reported as viable based on the output of a flow cytometric Live/Dead assay. To gain a better understanding of microbial community structure in these samples, total DNA from 0.2 micron filters was amplified by PCR and used to construct bacterial and archaeal 16S rRNA gene libraries. A total of 84 complete (∼1400 bp) bacterial sequences were constructed and consolidated into 23 representative taxa that were submitted to NCBI (Accession #:MK682771-MK682793). A total of 35 partial (∼850 bp) archaeal sequences were consolidated into 3 representative taxa that were also submitted to NCBI (Accession #: MK675956-MK675958). Bacterial communities were dominated by Proteobacteria (81% - a mix of Alphaproteobacteria, Betaproteobacteria, Deltaproteobacteria, and Gammaproteobacteria), Acidobacteria (11%), and Firmicutes (8%) (Supplementary Figure S1). Among the archaea, clones were distributed between Euryarchaeota (71%) predominantly a species related to *Methanothermobacter thermautotrophicus* and *Methanococcus igneus*, and Crenarchaeota (29%) (Supplementary Figure S2). Close relatives of strain DRI-13^T^ were not detected in these libraries.

### Isolate Properties and Identification

**FIGURE 2:**
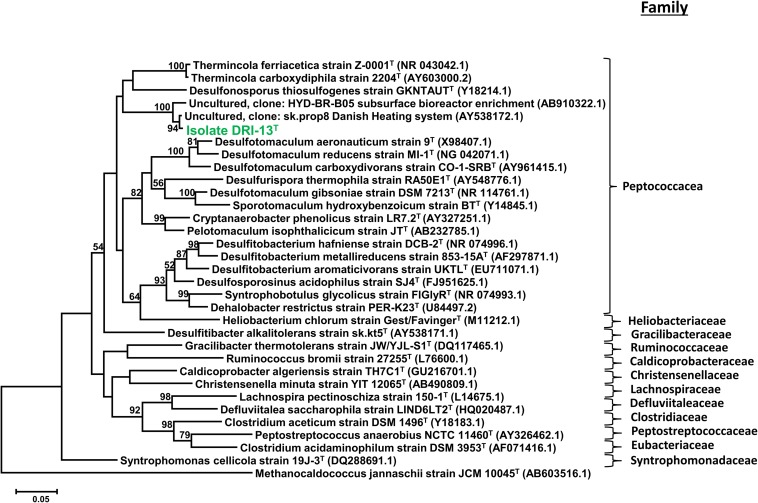
Taxonomic placement of isolate DRI-13^T^ by full length 16S rRNA genes aligned by SILVA (v1.2.11), and maximum-likelihood method based on 1,000 permutations of all bacterial families with nomenclature standing within the class *Clostridiales*. The scale bar represents 5% identity dissimilarity between sequences.

Strain DRI-13^T^ was purified from a 45°C anaerobic enrichment in a synthetic groundwater medium supplemented with 0.1% wt/vol peptone as the sole carbon and energy source. After 3 weeks of isolation techniques (see methods), the DRI-13^T^ colonies were 0.5 mm in diameter, circular (with entire margins), glossy, and convex. The resulting isolate was a Gram-stain-positive bacillus (∼2–6 μm long and ∼0.5 μm wide) which reached a density of ∼10^6^ cells/mL in culture. The strain was motile and was observed by phase contrast microscopy to form a central endospore. Phylogenetic analysis of the full-length 16S rRNA gene (GenBank accession no. KR014122) indicated that DRI-13^T^ possessed 90.0% similarity to *Pelotomaculum propionicicum* (Imachi et al., 2007) and *P. thermopropionicum* (Imachi et al., 2002) and 88.0% similarity to *Desulfotomaculum gibsoniae* (Kuever et al., 1999). The most closely related 16S rRNA gene sequences in available databases affiliated with as-yet uncultured bacteria discovered from subsurface environments in Denmark (99.0% similarity, AY538172.1), Australia (97.0% similarity, EU400652.1), and Japan (97.0% similarity, AB910322.1). By comparing the 16S rRNA gene sequence to selected type strains representing all families under the order *Clostridiales*, it appears strain DRI-13^T^ is a previously undescribed genus within the family *Peptococcacea* (Figure 2).

### Physiological Characterization of Strain DRI-13^T^

**FIGURE 3:**
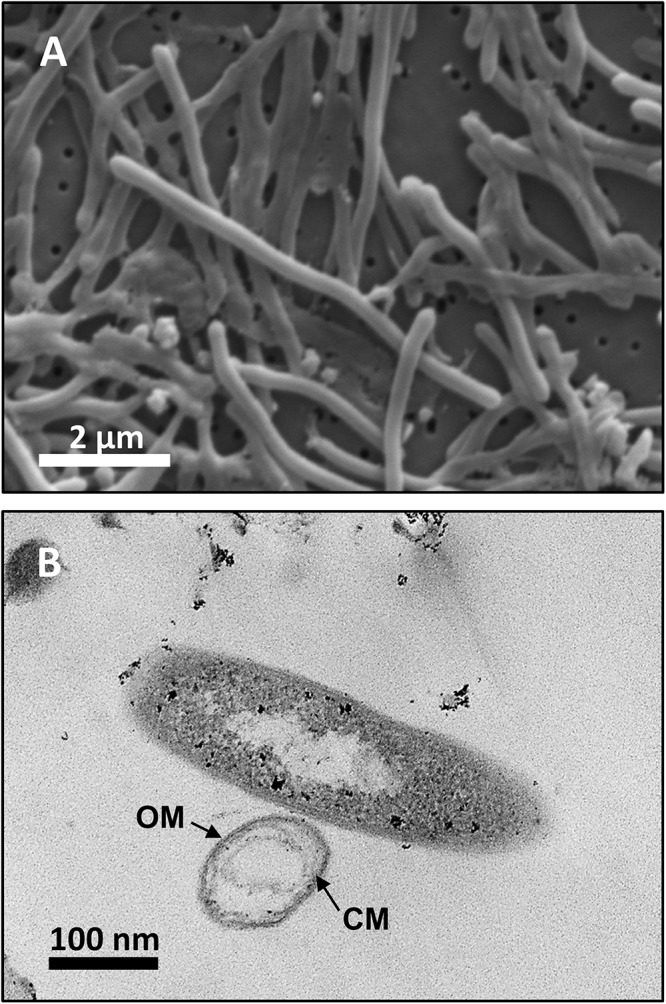
Microscopy images of strain DRI-13^T^: **(A)** scanning electron micrograph showing average 4–6 μm length morphology; **(B)** transmission electron micrograph showing outer membrane (OM) and cytoplasmic membrane (CM).

Analysis by SEM of isolate DRI-13^T^ revealed a distinctive thin rod-shaped morphology characterized by an average length of 4–6 μm and 0.5 μm width (Figure 3A). TEM revealed a distinct inner and outer membrane with an electron-dense matrix and what appears to be a vacuole (Figure 3B).

Strain DRI-13^T^ is a fastidious obligate anaerobe. The isolate grew poorly with 0.1% wt/vol casamino acids, 10 mM glucose, 0.1% wt/vol peptone, or 0.1% wt/vol yeast extract when used as the sole carbon and energy source. No growth was observed when supplemented with H_2_/CO_2_ (80:20), CO, formate, lactate, acetate, pyruvate, CH_4_, methanol, propionate, ribose, xylose, oxaloacetate, succinate, tartarate, butyrate, malate, citrate, aspartate, methionine, and valine. Stickland reactions of alanine/glycine, leucine/glycine, and glutamate/glycine also failed to support growth (Nisman, 1954). Likewise, accessory electron acceptors including nitrite, nitrate, sulfate, sulfite, thiosulfate, and elemental sulfur failed to stimulate growth. The only substrate tested that was successful in supporting growth was fumarate (Table 2).

**TABLE 2:** Carbon and electron utilization of DRI-13^T^ and closest relatives.

**Characteristic**	**DRI-13^T^**	***Pelotomaculum propionicicum* DSM 15578^1^**	***Pelotomaculum thermopropionicum* DSM 13744^2^**
Isolation source and depth	Nevada well U-3cn#5 at 0.7 km	Anaerobic sludge blanket reactor	Anaerobic sludge blanket reactor
Morphology	Rod	Rod	Rod
Cell size (μm)	0.5 × 6.0	1.0 × 3.0	0.7 × 2.0
Motility	Yes	No	No
Spore former	Yes	Yes	Yes
Spore location	Central	Central	Central
Optimal Temperature for growth (°C)	55	37	55
Temperature range (°C)	35–65	25–45	45–65
Optimal pH for growth (range)	8.0 (7.0–8.5)	7.0 (6.5–7.4)	7.0 (6.5–8.0)
NaCl limit of growth (mM)	100	85	68
Genome Sequenced	Yes	No	Yes
Doubling time (hours)	2.5	120	14.5
**Utilization of electron/carbon donors**			
H_2_/CO_2_	–	–	–
CO	–	ND	ND
Formate	–	–	–
Lactate	–	–	+^∗^
Acetate	–	–	–
Pyruvate	–	–	+
Methane	–	ND	ND
Methanol	–	–	–
Propionate	–	+	+^∗^
Ribose	–	–	–
Glucose	(+)	–	–
Xylose	–	–	–
Casamino acids	(+)	–	–
Peptone	(+)	–	+
Yeast Extract	(+)	–	+
Fumarate	+	–	–
Oxaloacetate	–	ND	ND
Succinate	–	–	–
Tartarate	–	ND	ND
Butyrate	–	–	–
Malate	–	–	–
Citrate	–	ND	ND
Aspartate	–	–	ND
Methionine	–	ND	ND
Alanine + glycine	–	–	ND
Leucine + glycine	–	–	ND
Glutamate + glycine	–	–	ND
Valine	–	ND	ND
**Utilization of electron acceptors**			
Fumarate	+	–	+
Nitrite	–	ND	ND
Nitrate	–	–	–
Sulfate	–	–	–
Thiosulfate	–	–	–
Sulfite	–	–	–
Inorganic Sulfur	–	–	–

*ND, not determined. (), limited growth observed over 2–3 weeks. ^∗^, only in co-culture with *Methanothermobacter themautotrophicus* strain DH^*T*^. −, no observed growth; +, growth was observed. ^1^Imachi et al. (2007). ^2^Imachi et al. (2002).*

**FIGURE 4:**
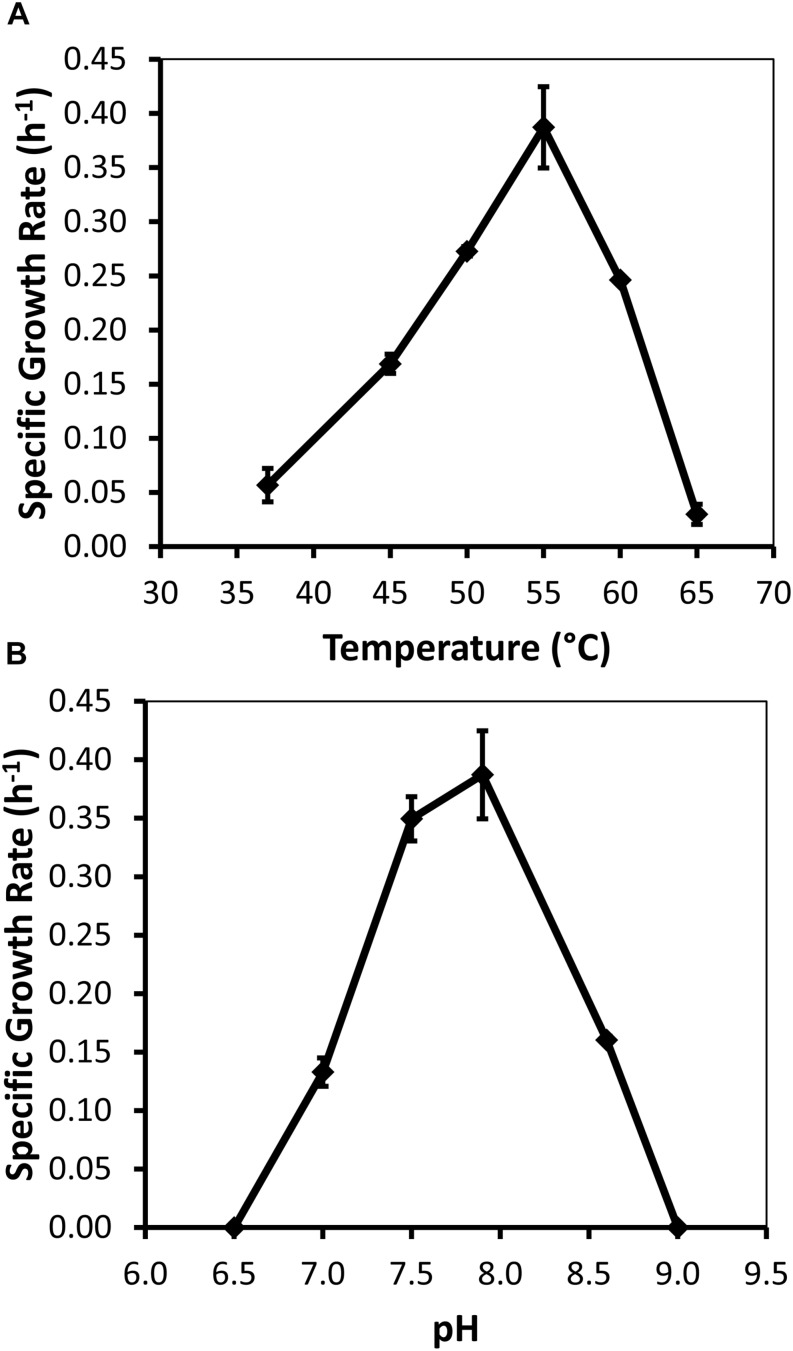
Growth characteristics of strain DRI-13^T^ for: **(A)** optimal temperature of 55°C; and **(B)** optimal pH of 8. Data points averaged from quadruplicate cultures using a standardized medium of salts with 10 mM fumarate.

**FIGURE 5:**
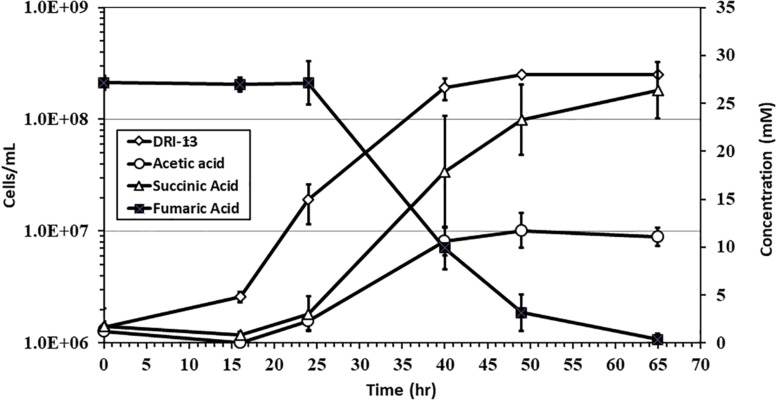
Growth of DRI-13^T^ on 30 mM fumarate and metabolic end products: open diamonds, cell density; filled squares, fumarate depletion; filled triangles, succinate production; filled circles, acetate production. Each data point is an average of triplicate samples with standard deviation error bars.

Specific growth rates of DRI-13^T^ during anaerobic respiration on fumarate display a maximal specific growth rate of 0.4 h^–1^ under optimal conditions of temperature and pH (55°C and pH 8.0) (Figures 4A,B). Metabolic analysis of DRI-13^T^ shows growth vs. substrate utilization experiment (30 mM fumarate) performed over 70 h (Figure 5). This experiment revealed an initial lag phase of 15–20 h, after which cellular growth commenced along with consumption of fumarate, reaching the lower limit of detection at 65 h. Temporally corresponding to the depletion of fumarate was the coincident production of succinate and acetate, which reached maxima at about 50 h. These experiments revealed a conversion rate of one equivalent of fumarate to approximately 0.83 and 0.33 equivalents of succinate and acetate.

Lipid analysis indicates that the major fatty acids (>10%) present for strain DRI-13^T^ are the branched-chain fatty acid iso-C_15__:__0_ (22.8%), straight-chain C_15__:__0_ (15.0%), C_16__:__0_ (12.0%), and C_14__:__0_ (11.6%), as compared to phylogenetically related bacteria (Table 3).

**TABLE 3:** Cellular fatty acid composition (%) of strain DRI-13^T^ compared with its closest phylogenetic neighbors.

**Fatty Acid**	**DRI-13^T^**	**SI^T^^1^**	**JT^T^^2^**	**LR7.2^T^^3^**	**Groll^T^^4^**
C_11__:__0_	–	–	–	–	1.9
Anteiso-C_13__:__0_	2.1	–	–	–	–
C_13__:__0_	1.3	–	–	–	–
iso-C_14__:__0_	–	–	–	–	1.6
C_14__:__0_	**11.6**	3.2	26	5.8	2.6
iso-C_15__:__1_ F	2.9	–	–	–	–
^∗^iso-C_15__:__1_ H/C_13__:__0_ 3OH	1.2	–	–	–	–
iso-C_15__:__0_	**22.8**	**76.4**	**28.7**	**13.7**	5.4
anteiso-C_15__:__0_	2.5	–	9.2	**18.9**	–
C_15__:__1_ω8*c*	2.1	–	–	–	–
C_15__:__1_ω7*c*	–	–	–	–	2.7
C_15__:__1_ω6*c*	1.5	–	–	–	–
C_16__:__0_ alde	–	–	–	–	2.2
C_15__:__0_	15	–	–	3.5	–
C_15__:__0_ dma	–	–	–	–	1.7
iso-C_16__:__0_	–	–	–	7.5	–
C_16__:__1_ω7*c*	–	–	–	–	3.3
C_16__:__1_ω9*c*	1.1	–	–	**13.7**	**15.1**
C_16__:__1_ω11*c*	–	–	–	–	3.6
^∗^C_16__:__1_ω7*c*/C_16__:__1_ω6*c*	5.8	–	–	–	–
C_16__:__0_	12	**10.7**	**16.7**	**12.5**	9.6
C_16__:__1_ω9*c* dma	–	–	–	–	4.6
C_16__:__0_ dma	–	–	–	–	6.7
iso-C_17__:__1_ ω7*c*	–	–	–	–	4.8
iso-C_17__:__1_ ω9*c*	4.9	2.9	–	–	–
C_17__:__0_ cyclo	–	–	–	–	2.7
iso-C_17__:__0_	1.8	–	–	5.7	–
anteiso-C_17__:__0_	–	–	–	7.3	–
C_17__:__1_ω8*c*	1.5	–	–	–	–
C_17__:__0_	1	–	–	–	–
C_18__:__1_ω9*c*	–	–	–	4.6	2
C_18__:__1_ω11*c*	–	–	–	–	3.3
C_18__:__1_ω13*c*	–	–	–	–	2.8
C_18__:__0_	3.6	6.7	7.1	4.2	5.6
C_18__:__1_ω11*c* dma	–	–	–	–	1.5

*Only fatty acids that represent ≥ 1% of the total fatty acids of at least one strain are shown. ^∗^Summed features represent groups of two FAs not separated by MIDI system. alde, aldehyde; dma, dimethyl acetal; cyclo, cyclopropane. ^1^*Pelotomaculum thermopropionicum* strain SI^*T*^. ^2^*Pelotomaculum terephthalicicum* strain JT^*T*^. ^3^*Cryptanaerobacter phenolicus* strain LR7.2^*T*^. ^4^*Desulfotomaculum gibsoniae* strain Groll^*T*^. Bold values indicate major FA that are >10%.*

### Genome Sequencing and Analysis

High-quality genomic DNA was extracted from isolate DRI-13^T^ for genome sequencing by the DOE Joint Genome Institute (JGI, JGI GOLD Gi0054453, NCBI Taxonomic ID 1449126, and Assembly #ASM74602v1). The completed draft genome of DRI-13^T^ is 3,649,665 bp, with an average G + C content of 45% and composed of 105 contigs (Supplementary Table S1). A total of 3,749 genes were identified, of which 3,671 (98%) are predicted to code for proteins (PCGs). Predicted function could be assigned to 2874 (77%) of the total number of PCGs, while 797 (21%) had no known functional prediction^2^. Genes were identified for assimilatory/dissimilatory sulfate and sulfite reduction, anaerobic fumarate respiration, tripartite ATP-independent secondary passive transport, endospore formation/germination, and flagella biosynthesis (Supplementary Table S2).

## Discussion

Within North America’s Basin and Range (B&R) physiographic province lies the Great Basin (Grayson, 2011), a hydrogeologically defined, internally draining province that comprises portions of the US states of California, Oregon, Idaho, Arizona, and most of Nevada. Fault-controlled flow of deep groundwater within this tectonic extensional zone results in hydrographic basins and sub-basins (Winograd and Thordarson, 1975, Winograd et al., 2005), with the subject of this study, the Death Valley Regional Flow System (DVRFS), being the largest. Borehole U-3cn#5 (Figure 1) intercepts a hydrologically confined lobe of the DVRFS’s and represents a novel window for deep biosphere study of the Basin and Range and extensional zones in general.

The fracture carbonate-hosted aquifer from which DRI-13^T^ was isolated possesses chemistries (e.g., circumneutral pH, relatively high sulfate, low TOC) and overall microbial community structure typical of subsurface fractured carbonate aquifers studied elsewhere (Table 1) (Hohnstock-Ashe et al., 2001; Farnleitner et al., 2005; Gihring et al., 2006; Pfiffner et al., 2006; Park et al., 2009). Nutrients, fixed organic carbon, and energy resources are all relatively limited in this environment. As with other fractured carbonate aquifers, it is unclear how microbial populations are sustained, especially with regard to carbon sources (Hohnstock-Ashe et al., 2001). In U-3cn#5, rRNA gene libraries reveal a microbial community composed of predicted thermophilic anaerobes from the domain Bacteria and Archaea (Supplementary Figures S1, S2). The geothermal/carbonate-controlled nature of this subsurface environment makes it a distinct environment compared to surrounding monitoring wells, which are emplaced in shallower lower temperature systems and other rock types (e.g., volcanics and alluvium), with one exception: borehole BLM-1. Located approximately 75 km South of U-3cn#5 in the discharge zone of the DVRFS, this latter well intersects anoxic geothermal waters (57°C) of the regional fractured carbonate aquifer. In spite of this distance, these two wells share similar bacterial and archaeal profiles, including the presence of important marker subsurface bacteria such as “*Candidatus* Desulforudis audaxviator,” *Desulfotomaculum putei*, and the archaeon *Methanothermobacter thermautotrophicus* (Chivian et al., 2008; Thomas et al., 2013; Sackett, 2018).

Enrichment cultivations from U-3cn#5 water samples in a strictly anaerobic minimal salts medium amended with peptone produced a slowly growing thermophilic bacillus after ∼1 month at 50°C. Sugar and short chain carbohydrate substrates did not promote growth (Table 2). While not directly detected in the subsurface environment of U-3cn#5, the citric acid cycle intermediate fumarate was the only substrate that enabled routine culturing of the microorganism. Isolation techniques yielded a pure culture which was given the strain name, DRI-13^T^. Analysis of the 1463 nucleotide 16S rRNA gene sequence revealed that DRI-13^T^ affiliates at the species level with uncultured clones from other geothermally influenced subsurface locations including Japan, Australia (unpublished), and Denmark (AB910322.1, EU400652.1, AY538172.1) (Kjeldsen et al., 2007; Baito et al., 2015). Within the family *Peptococcaceae*, DRI-13^T^ is most closely related (∼90.0% similarity to the 16S rRNA gene) to three type strains *Pelotomaculum propionicicum*, *P. thermopropionicum*, and *P. isophthalicicum*. This establishes DRI-13^T^ as a novel genus using the parameters defined by Yarza et al. (2014) (Figure 2). Of these phylogenetic relatives, only *P. thermopropionicum* share thermophilic characteristics and utilization of fumarate (Table 2). Unlike DRI-13^T^, *P. thermopropionicum* can utilize a wider range of substrates and is described under certain conditions to support a syntrophic interaction with the methanogen *Methanothermobacter thermoautotrophicus*. The most abundant archaeon in our U-3cn#5 rRNA gene libraries was *M. thermoautotrophicus* (71% of reads). *P. propionicicum* and *P. isophthalicicum* have been reported to have syntrophic interactions with other methanogens as well, though no interactions were established between DRI-13^T^ and an isolated wild type *M. thermoautotrophicum* from the borehole. Growth combinations only resulted in DRI-13^T^ becoming the majority within the culture bottle and causing no change in growth performance with or without *M. thermoautotrophicum* (not shown).

Examination of DRI-13^T^ by SEM indicates a bacillus-type morphology, averaging 6 μm in length and 0.5 μm width (Figure 3A). TEM reveals features of the outer membrane and cytoplasmic membrane, including what appears to be a vacuole (Figure 3B), though it is possible that the space within the cell may be an artifact from TEM preparation. If real, the presence of a vacuole may explain an apparent buoyant property of DRI-13^T^ cells requiring centrifugation times greater than 30 min at or above 15,000 × *g*, which is typical for very small cells or for cells with natural buoyancy. Lab-standard cells from the phylum *Firmicutes*, such as *Desulfotomaculum putei*, can be pelleted with less rigorous effort similar to *E. coli*. While not a feature noted in other subsurface microorganisms, it is conceivable that innate buoyancy control could be advantageous to fracture-dwelling subsurface microorganisms during planktonic growth.

Measureable DRI-13^T^ growth was observed to occur between 37°C (minimum) and 65°C (maximum). While the environmental temperature of U-3cn#5 was recorded at 44.7°C, the optimal temperature for DRI-13^T^ is 55°C (Figure 4A). Although not determined, the *in situ* temperature from the interval where DRI-13^T^ was obtained was likely higher than that recorded at the wellhead due to the long distance pumped water was lifted through presumably cooler conditions in the aquifer above the confining layer and ∼500 m of vadose zone. As might be expected for an organism adapted to strongly buffered carbonate aquifers, the pH tolerance of DRI-13^T^ is narrow, with observable growth occurring only between 7.0 and 8.5, and optimal growth at pH 8.0 (Figure 4B). As compared to close relatives, the optimal temperature is similar to *P. thermopropionicum* and *P. propionicicum* (55°C and 37°C). The growth rate of DRI-13^T^ appears to be substantially faster, doubling every 2.5 h, as opposed to 14.5 and 120 h for *P. propionicicum* and *P. thermopropionicum*, respectively (Imachi et al., 2002, 2007). This faster growth rate possibly reflects the independent lifestyle of DRI-13^T^ as opposed to the syntrophic associations of the others. In addition, cells cultured beyond stationary phase (approximately 48 h) exhibit endospore formation, as observed by microscopy and the presence of endospore related genes (Supplementary Table S2). While no discernable cellular movement was consistently observed, DRI-13^T^ genome appears to have flagella related genes as well (Supplementary Table S2).

Lipid analysis indicates that the major fatty acids (>10%) for strain DRI-13^T^ are the branched-chain fatty acid iso-C_15__:__0_ (22.8%) along with the straight-chain C_15__:__0_ (15.0%), C_16__:__0_ (12.0%) and C_14__:__0_ (11.6%). The higher proportion of the branched-chain iso-C_15__:__0_ in strain DRI-13^T^ is also characteristic of its nearest neighbor, *P. thermopropionicum* strain SI^T^ in which iso-C_15__:__0_ represents the majority of all fatty acids at 76.4% (Imachi et al., 2002), and in *P. terephthalicicum* strain JT^T^ (28.7%) (Qiu et al., 2006), *Cryptanaerobacter phenolicus* strain LR7.2^T^ (Juteau et al., 2005), and *Desulfotomaculum gibsoniae* Groll^T^ (Kuever et al., 1999) (Table 3). While the overall fatty acid methyl ester (FAME) profile for strain DRI-13^T^ clearly differentiates it from its nearest phylogenetic neighbors, the significant percentage of the straight-chain fatty acid C_15__:__0_ is a defining characteristic, as it is not present in other closely related species, with the exception of *C. phenolicus* strain LR7.2^T^, in which it is only a minor component (Juteau et al., 2005). Interestingly, C15 lipids were not detected in any of 44 fracture and mine water samples from a comprehensive survey of environmental lipids in the ultradeep mines of South Africa (Pfiffner et al., 2006). Another defining characteristic is the detection of C_16:1ω 7c/_C_16:1ω 6c_ (5.8%), which is not present in other phylogenetic neighbors such as *C. phenolicus* strain LR7.2^T^ (Juteau et al., 2005), and *D. gibsoniae* strain Groll^T^ (Kuever et al., 1999). C_16:1ω 7c_ was detected in the South African study, but statistically corresponded with mine service water samples rather than pristine fracture water.

Fumarate catabolism was typical as described previously in other Firmicutes (Dorn et al., 1978; Imachi et al., 2002). High-pressure liquid chromatography (HPLC) detected the removal of fumarate and the concomitant production of succinate and acetate. This supports the idea that fumarate is both being metabolized and used as an electron acceptor in anaerobic respiration (Figure 5). No other observable products such as oxalate, tartrate, lactate, malate, citrate, or propionate were observed by HPLC. Headspace pressure in culture bottles remained stable (∼1 bar) with no obvious over-pressurization. The source of fumarate in the subsurface is unclear. Gelling agents associated with drilling can be a source of fumarate (Borchardt, 1989). However, in this case residue from the drilling of U-3cn#5 may be unlikely given the borehole age (1965) and its flushing with multiple millions of liters of water pumped over the years. Samples of 1 g fractured carbonate from U-3cn#5 (NNSS Core Library), aseptically crushed, extracted with 5 mL boiling water, concentrated, and analyzed on HPLC, did not contain detectable levels of fumarate (data not shown). It is more likely that DRI-13^T^ is cryptically biodegrading the waste products or endogenous decay derived from other members of the microbial community (Béranger et al., 2006). Since DRI-13^T^ does not seem to be a dominant microbial community member (Supplementary Figure S1), it is possible that its relatively low representation is the result of occupying a narrow niche focusing on fumarate respiration or is a relic from a period after drilling when fumarate might have been present.

Assembly of the DRI-13^T^ draft genome from a single library prep resulted in 105 contigs. Complete coverage of the genome failed in regions where repetitive intergenic sequences (mobile elements), hypothetical proteins, or clustered regularly interspaced short palindromic repeats (CRISPR) arrays were present. The estimated genome size is 3,649,665 with a G + C content of 45%. This is comparable to close relatives with sequenced genomes such as *D. gibsonia* (4.85 Mbp) and *P. thermopropionicum* (3.02 Mbp). Distribution of nucleotides and genes across the 105 contigs range from 1,045 to 193,268 bases (average 34,759 bases per contig), with 2 to 203 annotated genes (average 36 genes per contig). The JGI/IMG annotation gene calling algorithms predict 3,749 genes in DRI-13^T^ with 77% of them functionally assigned (Supplementary Table S1). Analysis of the metabolic capability of the DRI-13^T^ genome indicates core genes for glycolysis/gluconeogenesis, fatty acid biosynthesis, and amino acid metabolism. Homologs for assimilatory or dissimilatory sulfate and sulfite reduction (*dsrA*, *dsrB*, and *dsrD*) are present. However, sulfide production was never detected when sulfate was present in the media. Amino acid analysis of the *dsr* genes show an identity of 84% to 99% similarity to *Desulfitobacter alkalitolerans*, which was isolated from a Danish geothermal heating system, a site where an uncultured 16S rRNA gene with exact homology to DRI-13^T^ had been sequenced (Nielsen et al., 2006; Kjeldsen et al., 2007).

Examination of the anaerobic fumarate respiration pathway revealed that core fumarate catabolic and related genes are present within the genome. Some of the genes for fumarate respiration have undergone duplication generating paralogs (Supplementary Table S2). The metabolic pathway for fumarate respiration converts three fumarate molecules to one acetate and two succinate molecules (Supplementary Figures S3A,B). The gene for the enzyme fumarase is present in the genome and can reversibly convert fumarate by hydration to malate or malate to fumarate by dehydration. The decarboxylation of the malate molecule to pyruvate is completed by NADP dependent malate dehydrogenase (oxaloacetate-decarboxylating), generating carbon dioxide and reductive potential. The further oxidation of pyruvate to acetyl-CoA is accomplished by pyruvate ferredoxin oxidoreductase, generating carbon dioxide and reduced ferredoxin (Thauer et al., 1977). The final reduction of acetyl-CoA with succinate to acetate occurs by succinyl-CoA:acetate CoA-transferase. Concurrently, two molecules of fumarate can be reduced to succinate by succinate dehydrogenase which was also annotated. Generation of ATP by coupling substrate-level phosphorylation is achieved by succinyl-CoA synthetase. DRI-13^T^ generates a proton motive force for ATP synthesis through a NADH ubiquinone-oxidoreductase (Nuo Complex 1) and membrane bound Ni-Fe hydrogenase-4 (Marreiros et al., 2013; Schut et al., 2016; Yu et al., 2018). The FPO_F_ complex allows versatility of energy conservation among anaerobes and is perhaps a recent adaptation to environments rich in sulfide and at an interface with oxygen (e.g., buoyancy), similar to water chemistry conditions found in monitoring well U-3cn#5 (Schut et al., 2016). It is not known what molecule transports electrons to the hydrogenase-4 Ni-Fe complex. Interestingly, seven copies of electron transfer flavoprotein (EtfAB) and quinone oxidoreductase (FixC) were annotated within the genomes, suggesting capacity for high potential electron transfers in bifurcating energy conservation (Costas et al., 2017).

Secondary transporters dependent on substrate binding are ubiquitous in prokaryotes yet poorly characterized. Tripartite ATP-independent periplasmic (TRAP) transporters manage the passive transport of hydrocarbon molecules across the membrane for the cell to use as a source of carbon, electrons, and energy. Isolate DRI-13^T^ has fourteen TRAP 4TM/12TM genes present in its genome, six of which have fused transmembrane subsunits (Supplementary Table S2). TRAP transporters are proposed to be ‘ancient’ secondary transporters that are anchored in the membrane, taking advantage of sodium ion gradients to co-transport 4 carbon molecules from outside the cell wall into the cytoplasm (Rabus et al., 1999; Kelly and Thomas, 2001; Mulligan et al., 2011). The TRAP transporter’s DctP subunit is seemingly specific to particular solutes (Fischer et al., 2015). DRI-13^T^’s almost exclusive utilization of the dicarboxylate molecule fumarate seems complimentary to the TRAP transport system. It has been demonstrated that the psychrophilic microorganism *Psychrobacter arcticus* 273-4, exhibited lower growth rates when its TRAP genes were genetically knocked out and afterward fed fumarate (Bakermans et al., 2009). DRI-13^T^ has 41 TRAP annotated transporter genes, compared with only three TRAP genes found in *P. thermopropionicum.* As for annotated genes, 10.4% of DRI-13^T^ genome fall under the category of transporter (390/3749 genes) as compared to 8.6% (259/3018) for *P. thermopropionicum*. Three of the fusion TRAP proteins in DRI-13^T^ genome (WP_034425417.1, WP_051965735.1, and WP_51966221.1) have 41-47% amino acid identity to homologs in the deep subsurface bacterium *Ca.* D. audaxviator (Chivian et al., 2008). The ‘investment’ of DRI-13^T^ genome with ATP independent secondary transporters potentially reveals a methodology of how subsurface microbes manage minimal energy metabolic activities in order to survive in oligotrophic environments. This is seemingly true for *Alphaproteobacteria* SAR11 that were sequenced from the Sargasso Sea, where TRAP transporters were found to be abundant (Morris et al., 2002; Mulligan et al., 2011).

Spore formation and germination genes were annotated in the genome as well (Brown et al., 1994; Browne et al., 2016). Isolate DRI-13^T^ appears to have 37 spore-related genes (Supplementary Table S2), as compared to microorganisms whose closest amino acid similarities to the *GerA* gene are *Bacillus camelliae*, which possesses 77 spore-related genes, and *Desulfonispora thiosulfatigenes*, which has 29 spore-annotated genes. Interestingly, the number of spore-related genes does not appear to differentiate microorganisms originating from surface and subsurface environments. For example, subsurface microorganisms such as *Desulfotomaculum putei* (26 spore-related genes), *Ca.* D. audaxviator (8 spore-related genes), and *Bacillus subterraneus* (64 spore-related genes) can be compared to surface microorganisms such as *Caldicellulosiruptor obsidiansis* (11 spore-related genes), and *Clostridium difficile* BI1 (17 spore related genes).

## Conclusion

The thermophilic, anaerobic, fumarate-respiring bacterium DRI-13^T^ was successfully isolated from geothermal water from the Lower Carbonate Aquifer (LCA) of the Death Valley Regional Flow System (DVRFS) collected from fractures at 863 – 923 mbls. Physiological and molecular analysis of strain DRI-13^T^ revealed it to be a new species within a novel genus of the Family *Peptococcaceae*. Microbial community analysis via 16S rRNA gene libraries of *Archaea* and *Bacteria* reveal a community consistent with other anoxic, rock-hosted subsurface aquifers. However, DRI-13^T^ was not present in these samples as a major community member. This study represents one of the first descriptions of indigenous life from the LCA of the DVRFS, and DRI-13^T^ is the first characterized microorganism from this deep subsurface extensional zone habitat. The apparent obligate reliance upon fumarate by this organism is relatively unusual and of uncertain significance in a deep biosphere context. Here we introduce DRI-13^T^ as a representative microorganism from the terrestrial deep biosphere and propose the name *Thermoanaerosceptrum fracticalcis* DRI-13^T^ gen. nov. sp. nov.

### Description of *Thermoanaerosceptrum* gen. nov.

*Thermoanaerosceptrum* gen. nov. (Ther.mo.an.a.e.ro.scep’trum. Gr. adj. *thermos*, hot; Gr. prefix an-, not; Gr. n. *aer* air; Gr. neut. n. *skeptron* staff; N.L. neut. n. *Thermoanaerosceptrum* a hot anaerobic staff).

Cells are rod-shaped. Cell wall is Gram-positive type. Central endospores are observed. Thermophilic. Obligate anaerobic chemoorganoheterotroph. Fermentation end products are succinate and acetate. The major fatty acids (>10%) were iso-C_15:0_, C_15:0,_ C_16:0_ and C_14:0_.

The type species is *Thermoanaerosceptrum fracticalcis.*

### Description of *Thermoanaerosceptrum fracticalcis* sp. nov.

(frac.ti.cal’cis. L. part. adj. *fractus*, broken; L. n. *calx*, -cis, limestone; N.L. gen. n. *fracticalcis*, of broken limestone, referring to the origin of the type strain).

Cells are long, straight rods averaging 6 μm long and 0.5 μm wide, occurring singly. The cell wall is Gram-positive type, with central endospores observed. Optimal growth temperature is 55°C, with a maximum of 65°C and minimum of 35°C. Optimal pH is 8.0 with a range of 7.0–8.5. Obligate anaerobic chemoorganoheterotroph that utilizes fumarate as a sole carbon source and electron donor/acceptor for growth. Glucose, casamino acids, peptone, and yeast extract can be weakly utilized as electron/carbon donors. Fermentation end products are succinate and acetate. The estimated genome size of DRI-13^T^ is 3,649,665 bp, of which 87.3% are coding regions. The major fatty acids (>10%) were iso-C_15__:__0_, C_15__:__0__,_ C_16__:__0_ and C_14__:__0_.

The type strain is DRI-13^T^ (DSM 100382^T^ = ATCC TSD-12^T^), which was isolated from a terrestrial deep biosphere aquifer located in the US Great Basin. The DNA G + C content of the type strain is 45.2 mol%.

## Data Availability Statement

The datasets generated for this study can be found in the JGI/IMG and NCBI.

## Author Contributions

SH-B designed the research project, organized the contributions of all other authors, conducted the experiments, collected the data, and led the writing effort. LS screened the first enrichments and initiated the isolation of microorganism. MZ, TO, and BL provided the geochemical data on U-3cn#5. MC and PL performed and interpreted the lipid analysis. JG and IN performed HPLC and submitted the Illumina genome sequencing analysis. CR contributed to the experimental design and provided the hydrogeological and NNSS logistical expertise. DM secured the site access, collected the samples, secured the major funding, and co-wrote the manuscript.

## Conflict of Interest

The authors declare that the research was conducted in the absence of any commercial or financial relationships that could be construed as a potential conflict of interest.

## Footnotes

1 http://www.bacterio.net/index.html

2 https://img.jgi.doe.gov
