# Supplementary material for: Thermoanaerosceptrum fracticalcis gen. nov. sp. nov., a Novel Fumarate-Fermenting Microorganism From a Deep Fractured Carbonate Aquifer of the US Great Basin
Source: Front Microbiol. 2019 Sep 27;10:2224. doi: 10.3389/fmicb.2019.02224 (PMC6776889; doi:10.3389/fmicb.2019.02224)
Supplement: Supplementary file 1 [file Data_Sheet_1.PDF]

Thermoanaerosceptrum fracticalcis gen. nov. sp. nov., a novel fumarate-fermenting microorganism from a deep fractured carbonate aquifer of the US Great Basin

Scott D. Hamilton-Brehm[1,2*], Laura E. Stewart[3], Mavrik Zavarin[4], Matt Caldwell[7], Paul Lawson[7], Tullis C. Onstott[5], Joseph Grzymski[1], Iva Neveux[1], Barbara Sherwood Lollar[6], Charles E. Russell[8], Duane P. Moser[1,8~~8~~*]

[1]Division of Earth and Ecosystems Sciences, Desert Research Institute, Las Vegas, NV, USA

[2]Department of Microbiology, Southern Illinois University Carbondale, Carbondale, IL, USA

[3]Madison College Area Technical College, Madison, WI, USA

[4]Lawrence Livermore National Laboratory, Livermore, CA, USA

[5]Department of Geosciences, Princeton University, Princeton, NJ, USA

[6]Department of Earth Sciences, University of Toronto, Toronto, Ontario, Canada

[7]Department of Microbiology and Plant Biology, University of Oklahoma, Norman, OK, USA

[8]Division of Hydrologic Sciences, Desert Research Institute, Las Vegas, NV, USA

*Corresponding authors: E-mail Scott.Hamilton-Brehm@siu.edu and Duane.Moser@dri.edu

SUPPLEMENTAL FIGURES

Supplemental Figure 1. Consolidated bacterial library clones from borehole U-3cn#5 with accession numbers. SILVA alignment of full length Bacteria 16S rRNA gene sequences, maximum-likelyhood method, 1000 bootstrap.

Supplemental Figure 2. Consolidated archaeal library clones from borehole U-3cn#5 with accession numbers. SILVA alignment of full length Bacteria 16S rRNA gene sequences, maximum-likelyhood method, 1000 bootstrap.

Supplemental Figure 3. Outline of fumarate fermentation metabolic pathway in isolate DRI-13^{T}. Key enzymes are numbered 1- 6 with annotated names: A) Energy conservation pathway of fumarate to acetate, (1) Fumarase, (2) Malate dehydrogenase, (3) Pyruvate ferredoxin oxidoreductase, (4) Succinyl-CoA:acetate CoA-transferase, and (5) synthetase Succinyl-CoA. B~~A~~) Complex II respiration (6) Fumarate reductase.

SUPPLEMENTAL TABLES

Supplemental Table S1: Genome Characterization of DRI-13^T

Feature	Value	% of Total
Contigs	105	-
Genome size (bp)	3,649,665	-
G+C %	45%	-
Total number of genes	3,749	100%
Protein coding genes	3,671	98%
Protein coding gene with predicted function	2,876	77%
Protein coding gene without predicted function	795	21%
RNA genes	78	2%
Mobile elements	33	1%

Supplemental Table S2: Fumarate metabolism, hydrogenases, TRAP, germination, and flagella associated genes in DRI-13^T

Enzyme	Accession	JGI/IMG Gene ID	Gene Product Name
Fumarase	WP_034421165.1	2574347304	Fumarase, class I alpha subunit
	WP_034421167.1	2574347305	Fumarate hydratase subunit beta, Fe-S
	WP_034421812.1	2574347685	fumarate hydratase subunit alpha
	WP_034421810.1	2574347684	Fumarate hydratase subunit beta, Fe-S
Malate Dehydrogenase	WP_034421163.1	2574347303	Malate dehydrogenase (oxaloacetate-decarboxylating)
	WP_034421809.1	2574347683	Malate dehydrogenase (oxaloacetate-decarboxylating)
Pyruvate Dehydrogenase	WP_034420388.1	2574346765	Pyruvate dehydrogenase E2 (dihydrolipoamide acetyltransferase)
	WP_034420389.1	2574346766	Pyruvate dehydrogenase E1 beta subunit
	WP_034420390.1	2574346767	Pyruvate dehydrogenase E1 alpha subunit
	WP_034421312.1	2574347401	Pyruvate dehydrogenase E2 (dihydrolipoamide acetyltransferase)
	WP_034421313.1	2574347403	Pyruvate dehydrogenase E1 beta subunit
	WP_034421314.1	2574347404	Pyruvate dehydrogenase E1 alpha subunit
	WP_034425279.1	2574349592	Pyruvate dehydrogenase E1 alpha subunit

	WP_034425281.1	2574349593	Pyruvate dehydrogenase E1 beta subunit
	WP_034425282.1	2574349594	Pyruvate dehydrogenase E2 (dihydrolipoamide acetyltransferase)
Pyruvate ferredoxin oxidoreductase	WP_034421201.1	2574347329	Pyruvate:ferredoxin (flavodoxin) oxidoreductase, homodimeric
	WP_081908275.1	2574349291	Pyruvate:ferredoxin (flavodoxin) oxidoreductase, homodimeric
	WP_034422094.1	2574347868	pyruvate ferredoxin oxidoreductase, beta subunit
	WP_034422096.1	2574347869	pyruvate ferredoxin oxidoreductase alpha subunit
	WP_051965705.1	2574347870	pyruvate ferredoxin oxidoreductase delta subunit
	WP_034422098.1	2574347871	pyruvate ferredoxin oxidoreductase, gamma subunit
Succinyl-CoA:acetate CoA-transferase	WP_034423858.1	2574348909	Succinyl-CoA:acetate CoA-transferase
Fumarate Reductase	WP_034421168.1	2574347306	Fumarate reductase subunit A
	WP_034421170.1	2574347307	Fumarate reductase Fe-S subunit B
	WP_034421171.1	2574347308	heterodisulfide reductase subunit C
Succinyl-CoA synthetase	WP_034422243.1	2574347956	Succinyl-CoA synthetase, alpha subunit

**Enzyme**	**Accession**	**JGI/IMG Gene ID**	**Gene Product Name**
**Hydrogenase 4**			
subunit A	WP_034423986.1	2574348958	HyfA, Fe-S-cluster-containing hydrogenase
subunit B	WP_034423988.1	2574348959	HyfB, Multisubunit Na+/H+ antiporter, MnhD subuni
subunit C	WP_034423991.1	2574348960	HyfC family protein, NADH dehydrogenase
subunit E	WP_034423993.1	2574348961	HyfE, NADH-ubiquinone/plastoquinone oxidoreductase
subunit F	WP_034423995.1	2574348962	HyfF, Hydrogenase subunit F
subunit G	WP_034423997.1	2574348963	HycE1 (hydrogenase III-G), HycE2 (NiFe, hydrogenase III)
subunit H	WP_034423999.1	2574348964	hyfH hydrogenase-4 component H, NuoI
**NADH dehydrogenase**			

subunit A	WP_034422816.1	2574348282	NuoA, NADH-ubiquinone/plastoquinone oxidoreductase
subunit B	WP_034422814.1	2574348281	NuoB, NADH-quinone oxidoreductase, B subunit
subunit C	WP_034422813.1	2574348280	NuoC, NADH-quinone oxidoreductase subunit C
subunit D	WP_081908180.1	2574348279	NuoD, NADH:ubiquinone oxidoreductase
subunit H	WP_034422809.1	2574348278	NuoH, NADH:ubiquinone oxidoreductase subunit H
subunit I	WP_034422807.1	2574348277	NuoI, NADH dehydrogenase, 4Fe-4S dicluster domain
subunit J	WP_034422804.1	2574348276	NuoJ, NADH-ubiquinone/plastoquinone oxidoreductase
subunit K	WP_034422802.1	2574348275	NuoK, NADH:ubiquinone oxidoreductase subunit K
subunti L	WP_034422801.1	2574348274	NuoL, NADH:ubiquinone oxidoreductase subunit 5 (chain L) / Multisubunit Na+/H+ antiporter, MnhA subunit
subunit M	WP_051965824.1	2574348273	NuoM, proton translocating NADH:ubiquinone oxidoreductase subunit 4 (chain M)
subunit N	WP_034422799.1	2574348272	NuoN, NADH:ubiquinone oxidoreductase subunit 2 (chain N)
**Ferredoxin hydrogenase**			
Lrg subunit	WP_034423748.1	2574348857	Large subunit, [FeFe] hydrogenase, group B1/B3

Membrane bound protein	Accession	JGI/IMG Gene ID	Gene Product Name
**Tripartite ATP-Independent periplasmic (TRAP) transporters**			
TRAP transporters	WP_081908004.1	2574346542	TRAP-type C4-dicarboxylate transport system, DctQ
	WP_034420118.1	2574346543	tripartite ATP-independent transporter solute receptor, DctP family
	WP_051965502.1	2574347095	tripartite ATP-independent transporter solute receptor, DctP family
	WP_051965505.1	2574347096	C4-dicarboxylate transporter, DctQ subunit
	WP_034420828.1	2574347097	C4-dicarboxylate transporter, DctM subunit
	WP_051965569.1	2574347258	tripartite ATP-independent transporter solute receptor, DctP family
	WP_034421112.1	2574347259	C4-dicarboxylate transporter, DctQ

		subunit
WP_034421113.1	2574347260	C4-dicarboxylate transporter, DctM subunit
WP_034421346.1	2574347385	C4-dicarboxylate transporter, DctM subunit
WP_051965591.1	2574347386	TRAP-type C4-dicarboxylate transport system, DctQ
WP_034421349.1	2574347387	tripartite ATP-independent transporter solute receptor, DctP family
WP_034421638.1	2574347587	C4-dicarboxylate transporter, DctM subunit
WP_051965642.1	2574347588	C4-dicarboxylate transporter, DctQ subunit
WP_034421667.1	2574347589	C4-dicarboxylate-binding protein DctP
WP_034421990.1	2574347794	C4-dicarboxylate transporter, DctM subunit
WP_034421991.1	2574347795	C4-dicarboxylate transporter, DctQ subunit
WP_034421992.1	2574347796	C4-dicarboxylate-binding protein DctP
WP_034422502.1	2574348103	C4-dicarboxylate transporter, DctM subunit
WP_051965765.1	2574348104	TRAP-type C4-dicarboxylate transport system, DctQ
WP_034422504.1	2574348105	tripartite ATP-independent transporter solute receptor, DctP family
WP_051965783.1	2574348146	C4-dicarboxylate transporter, DctQ subunit
WP_034422567.1	2574348147	C4-dicarboxylate transporter, DctM subunit
WP_051965785.1	2574348151	tripartite ATP-independent transporter solute receptor, DctP family
WP_051966079.1	2574349131	TRAP transporter, DctM subunit
WP_034424391.1	2574349132	TRAP-type C4-dicarboxylate transport system, DctQ
WP_034424393.1	2574349133	tripartite ATP-independent transporter solute receptor, DctP family
WP_034424736.1	2574349303	tripartite ATP-independent transporter solute receptor, DctP family
WP_034424738.1	2574349304	C4-dicarboxylate transporter, DctQ subunit
WP_034424763.1	2574349305	C4-dicarboxylate transporter, DctM subunit
WP_034421688.1	2574347611	TRAP transporter, 4TM/12TM fusion protein

Enzyme	Accession	JGI/IMG Gene ID	Gene Product Name
	WP_034421690.1	2574347612	C4-dicarboxylate ABC transporter substrate-binding protein
	WP_051965692.1	2574347785	TRAP transporter, 4TM/12TM fusion protein
	WP_081908132.1	2574347784	hypothetical protein (uncharacterized periplasmic component)
	WP_034421984.1	2574347788	TRAP transporter, 4TM/12TM fusion protein
	WP_034421981.1	2574347786	hypothetical protein (uncharacterized periplasmic component)
	WP_051965735.1	2574347931	TRAP transporter, 4TM/12TM fusion protein
	WP_034422209.1	2574347932	hypothetical protein (uncharacterized periplasmic component)
	WP_051965979.1	2574348828	TRAP transporter, 4TM/12TM fusion protein
	WP_034423708.1	2574348829	hypothetical protein (uncharacterized periplasmic component)
	WP_051966221.1	2574349665	TRAP transporter, 4TM/12TM fusion protein
	WP_081908318.1	2574349666	hypothetical protein (uncharacterized periplasmic component)

**Enzyme**	**Accession**	**JGI/IMG Gene ID**	**Gene Product Name**
**Sporulation and germination genes**			
Spore germination proteins	WP_081908054	2574347058	spore germination protein KA
	WP_081908055	2574347059	Spore germination B3/ GerAC like, C-terminal
	WP_034420769	2574347061	Spore germination protein
	WP_034421156	2574347296	spore germination protein KB
	WP_051965559	2574347297	spore germination protein KA
	WP_081908075	2574347298	spore germination protein KC
	WP_051965681	2574347721	Spore germination protein YaaH
	WP_034422138	2574347844	spore germination protein KB
	WP_034422068	2574347845	spore germination protein KC
	WP_034422070	2574347846	spore germination protein KA
	WP_081908226	2574348847	Sporulation and spore germination
	WP_081908227	2574348848	spore germination protein KC
	WP_034423743	2574348849	Spore germination protein

	WP_051965987	2574348850	spore germination protein KA
	WP_081908283	2574349356	spore germination protein KA
	WP_034424854	2574349357	spore germination protein (amino acid permease)
	WP_034424855	2574349358	spore germination protein KC
	WP_081908302	2574349556	spore germination protein
	WP_034425710	2574349796	Spore germination protein gerPA/gerPF
	WP_034420617	2574346970	Spore coat polysaccharide biosynthesis protein SpsF, cytidylyltransferase family
	WP_051965576	2574347335	spore cortex biosynthesis protein YabQ
	WP_081908100	2574347440	spore maturation protein B
	WP_034421407	2574347441	spore maturation protein A
	WP_034421673	2574347598	Small, acid-soluble spore protein, alpha/beta type
	WP_034422533	2574348122	Uncharacterized spore protein YtfJ
	WP_034422636	2574348183	small acid-soluble spore protein H (minor)
	WP_034422871	2574348321	spore protease
	WP_034422872	2574348322	Small, acid-soluble spore protein, alpha/beta type
	WP_034422874	2574348323	Small, acid-soluble spore protein, alpha/beta type
	WP_034423150	2574348463	spore coat assemly protein
	WP_051965898	2574348494	Spore coat associated protein JA (CotJA)
	WP_081908201	2574348495	spore coat protein JB
	WP_034423203	2574348496	spore coat protein JC
	WP_034423273	2574348560	spore photoproduct lyase
	WP_034424315	2574349103	Small, acid-soluble spore protein, alpha/beta type
	WP_081908308	2574349611	small acid-soluble spore protein F (minor alpha/beta-type SASP)
	WP_034425965	2574349905	spore coat protein, CotS family
**Protein**	**Accession**	**JGI/IMG Gene ID**	**Gene Product Name**
**Flagellar assembly, hook, biosynthetic proteins**			

Flagellar proteins	WP_034419695	2574346264	flagellar basal-body rod protein FlgB
	WP_034419696	2574346265	flagellar basal-body rod protein FlgC
	WP_034419697	2574346266	flagellar hook-basal body complex protein FliE
	WP_034419699	2574346267	flagellar M-ring protein FliF
	WP_034419701	2574346268	flagellar motor switch protein FliG
	WP_051965357	2574346269	Flagellar biosynthesis/type III secretory pathway protein FliH
	WP_051965360	2574346274	flagellar basal-body rod modification protein FlgD
	WP_051965361	2574346275	flagellar operon protein
	WP_034419706	2574346276	flagellar hook protein FlgE
	WP_034419708	2574346277	flagellar protein FlbD
	WP_034419713	2574346280	flagellar FliL protein
	WP_034419715	2574346281	flagellar motor switch protein FliM
	WP_034419717	2574346282	flagellar motor switch protein FliN/FliY
	WP_081907973	2574346284	Flagellar biosynthesis protein, FliO
	WP_034419723	2574346285	flagellar biosynthetic protein FliP
	WP_034419725	2574346286	flagellar biosynthetic protein FliQ
	WP_034419726	2574346287	flagellar biosynthetic protein FliR
	WP_034419728	2574346288	flagellar biosynthetic protein FlhB
	WP_034419729	2574346289	flagellar biosynthesis protein FlhA
	WP_034419731	2574346290	flagellar biosynthesis protein FlhF
	WP_034419732	2574346291	flagellar biosynthesis protein FlhG
	WP_034419733	2574346292	c-di-GMP-binding flagellar brake protein YcgR, contains PilZNR and PilZ domains
	WP_034419739	2574346298	RNA polymerase sigma factor for flagellar operon FliA
	WP_034420343	2574346712	flagellar protein FliS
	WP_034420344	2574346713	flagellar hook-associated protein 2
	WP_034420345	2574346714	flagellar protein FlaG
	WP_034420351	2574346724	flagellar assembly factor FliW
	WP_034420353	2574346726	flagellar hook-associated protein 3 FlgL
	WP_034420354	2574346727	flagellar hook-associated protein 1 FlgK
	WP_034420357	2574346730	flagellar operon protein TIGR03826
	WP_034420599	2574346946	flagellar basal-body rod protein FlgG

WP_034420600	2574346947	flagellar basal-body rod protein FlgG
WP_051965471	2574346948	flagellar protein FlgJ
WP_051965633	2574347580	flagellar biosynthesis protein FlhG
WP_034424161	2574349042	flagellar biosynthesis protein
WP_034426101	2574349963	MinD-like ATPase involved in chromosome partitioning or flagellar assembly
