# Supplementary figures and images for: Thermoanaerosceptrum fracticalcis gen. nov. sp. nov., a Novel Fumarate-Fermenting Microorganism From a Deep Fractured Carbonate Aquifer of the US Great Basin
Source: Front Microbiol. 2019 Sep 27;10:2224. doi: 10.3389/fmicb.2019.02224 (PMC6776889; doi:10.3389/fmicb.2019.02224)

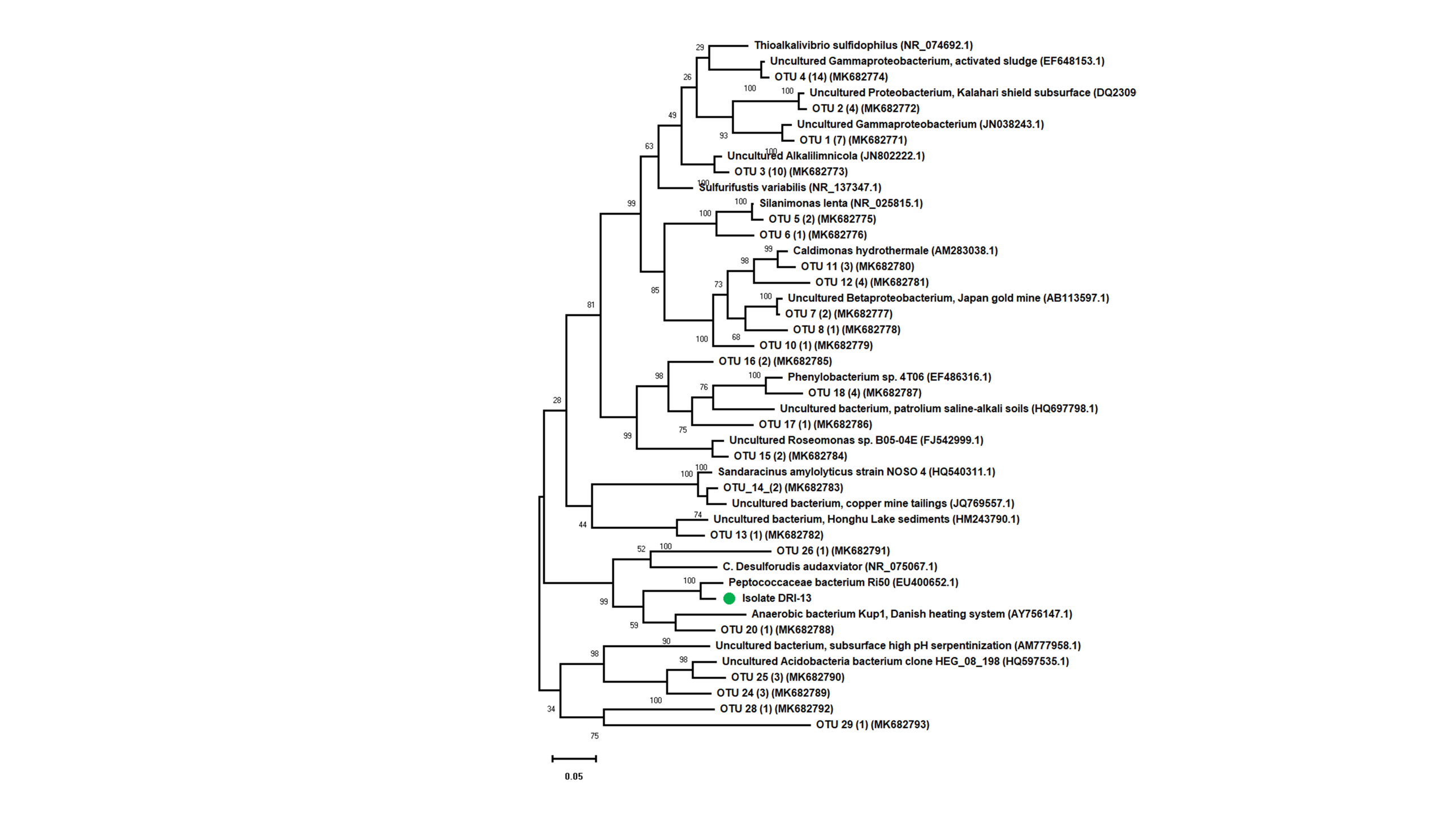

Supplement: Supplementary file 2 [file Image_1.TIF]

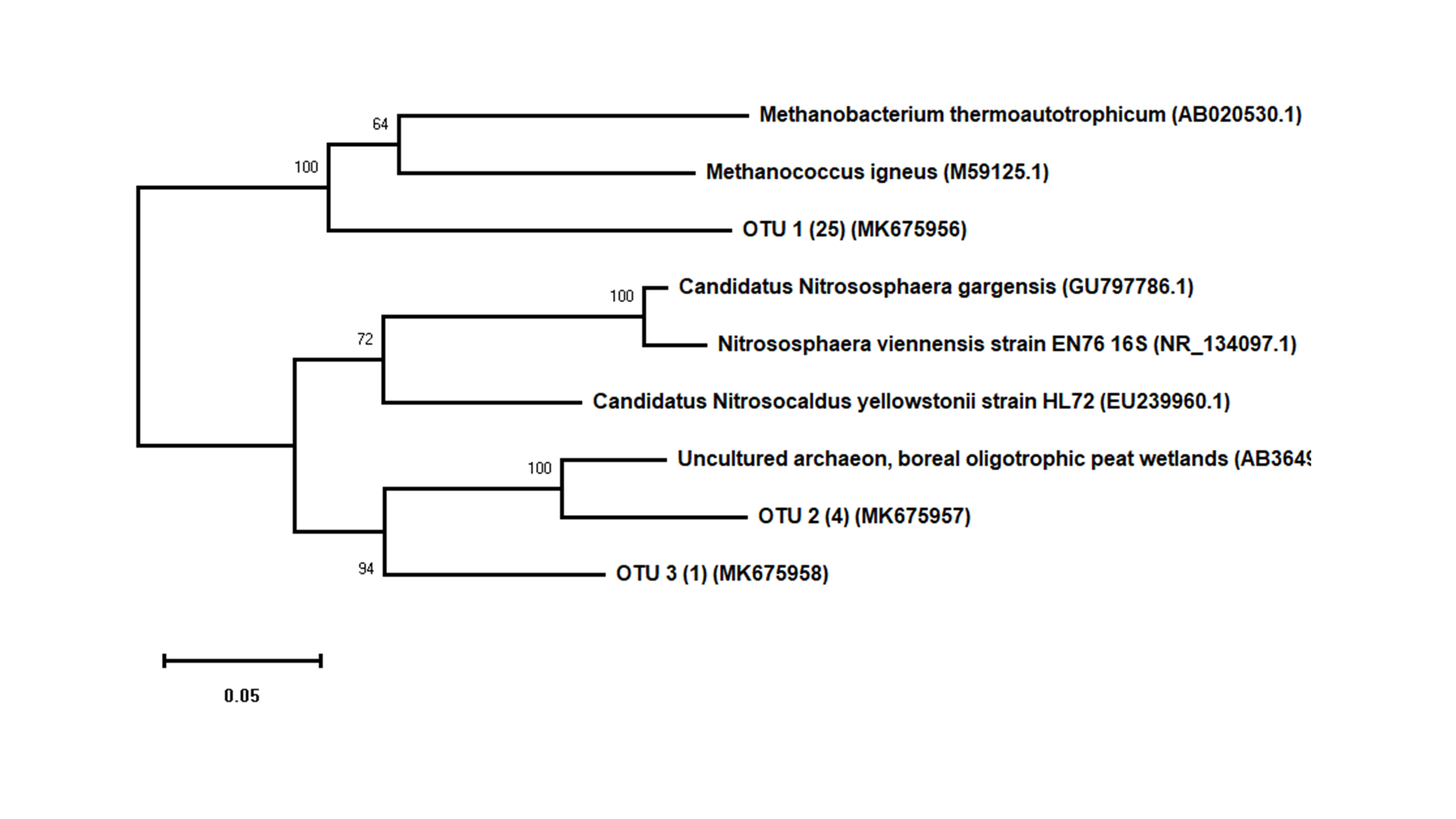

Supplement: Supplementary file 3 [file Image_2.TIF]

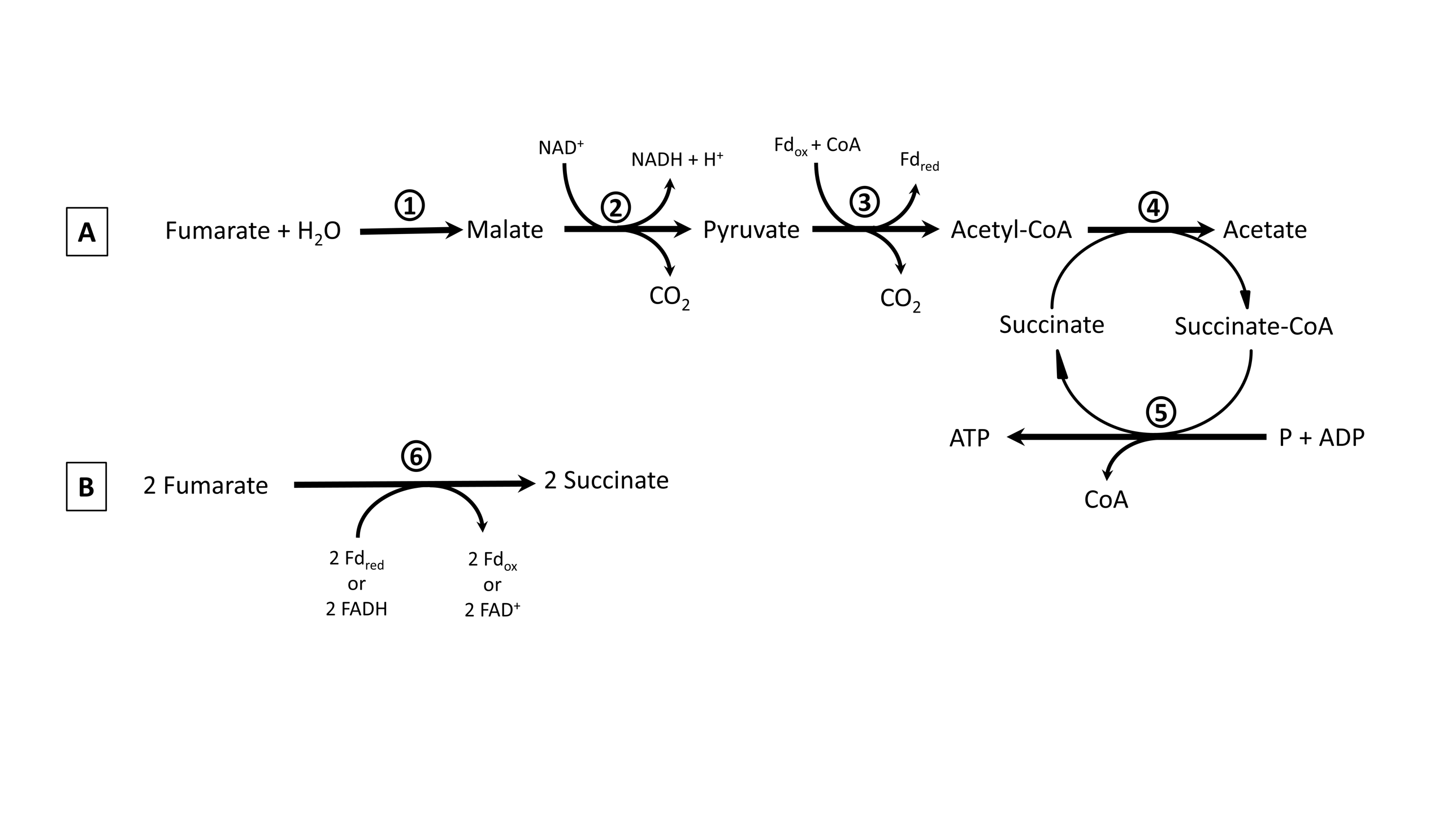

Supplement: Supplementary file 4 [file Image_3.TIF]
